# Targeting the phospholipase A2 receptor ameliorates premature aging phenotypes

**DOI:** 10.1111/acel.12835

**Published:** 2018-09-14

**Authors:** Audrey Griveau, Clotilde Wiel, Benjamin Le Calvé, Dorian V. Ziegler, Sophia Djebali, Marine Warnier, Nadine Martin, Jacqueline Marvel, David Vindrieux, Martin O. Bergo, David Bernard

**Affiliations:** ^1^ Centre de Recherche en Cancérologie de Lyon, Inserm U1052, CNRS UMR 5286, Centre Léon Bérard Université de Lyon Lyon France; ^2^ Department of Biosciences and Nutrition Karolinska Institutet Huddinge Sweden; ^3^ Centre International de Recherche en Infectiologie, Inserm U1111, CNRS, UMR5308, École Normale Supérieure de Lyon Université de Lyon Université Claude Bernard Lyon 1 Lyon France; ^4^ Present address: URBC‐NARILIS University of Namur Namur Belgium

**Keywords:** cellular senescence, p53, progeroid diseases, signaling

## Abstract

Hutchinson–Gilford progeria syndrome (HGPS) is a lethal premature aging that recapitulates many normal aging characteristics. This disorder is caused by mutation in the LMNA gene leading to the production of progerin which induces misshapen nuclei, cellular senescence, and aging. We previously showed that the phospholipase A2 receptor (PLA2R1) promotes senescence induced by replicative, oxidative, and oncogenic stress but its role during progerin‐induced senescence and in progeria is currently unknown. Here, we show that knockdown of PLA2R1 prevented senescence induced by progerin expression in human fibroblasts and markedly delayed senescence of HGPS patient‐derived fibroblasts. Whole‐body knockout of *Pla2r1* in a mouse model of progeria decreased some premature aging phenotypes, such as rib fracture and decreased bone content, together with decreased senescence marker. Progerin‐expressing human fibroblasts exhibited a high frequency of misshapen nuclei and increased farnesyl diphosphate synthase (FDPS) expression compared to controls; knockdown of PLA2R1 reduced the frequency of misshapen nuclei and normalized FDPS expression. Pamidronate, a FDPS inhibitor, also reduced senescence and misshapen nuclei. Downstream of PLA2R1, we found that p53 mediated the progerin‐induced increase in FDPS expression and in misshapen nuclei. These results suggest that PLA2R1 mediates key premature aging phenotypes through a p53/FDPS pathway and might be a new therapeutic target.

## 1 INTRODUCTION

The *LMNA* gene encodes lamin A and C proteins, which are located in the nuclear lamina where they contribute to rigidity and shape of the nuclear envelope and regulate chromatin organization and gene expression. Hutchinson–Gilford progeria syndrome (HGPS), a premature aging disease, is caused by a mutation in the *LMNA* gene, which leads to the activation of a cryptic splice donor site in exon 11 (Eriksson et al., 2003). The mutant prelamin A mRNA is then translated into progerin, an internally truncated protein that fails to undergo processing to mature lamin A and induces premature senescence (Goldman et al., 2004). ZMPSTE24 deficiency also leads to failure of maturing lamin A, to premature senescence and to progeria syndrome (Bergo et al., 2002).

The concept that cellular senescence contributes to pathologies linked to aging has been demonstrated over past few years. Indeed, eliminating senescent cells in mice with a progeroid syndrome delays some age‐associated disorders, while in wild‐type mice it reduces aging‐related diseases and extends lifespan (Baker et al., 2016, 2011 ). The list of age‐related diseases improved by delaying senescence or eliminating senescent cells is increasing and includes osteoporosis, type 2 diabetes, and atherosclerosis (Childs et al., 2016, 2017 ; Farr et al., 2017; Minamino et al., 2009). However, the role of cellular senescence in premature aging remains largely unclear.

Cellular senescence can be induced by replicative exhaustion, reactive oxygen species (ROS), genotoxic drugs, and ionizing radiation and results in stable proliferation arrest and the acquisition of a specific senescence‐associated secretory phenotype (SASP). In the context of aging and age‐related diseases, proliferation arrest is thought to limit organ renewal and the SASP is thought to alter the organization and function of tissues (Ovadya, & Krizhanovsky, 2014). We previously observed in primary human cells that constitutive expression of the phospholipase A2 receptor 1 (PLA2R1) induces premature senescence and that its knockdown delays telomere‐dependent senescence and stimulates escape from senescence induced by oxidative and oncogenic stress. PLA2R1 encodes a transmembrane protein that can bind to secreted phospholipase A2 (sPLA2) and some collagen and integrin isoforms; and may regulate cellular senescence through the activation of JAK/STAT signaling and the ERRα transcription factor (Augert et al., 2009, 2013; Bernard, & Vindrieux, 2014; Griveau et al., 2016; Vindrieux et al., 2013; Vindrieux et al., 2013).

These results raise the interesting question of whether PLA2R1 may contribute to premature aging. In this study, we used progerin‐expressing fibroblasts, HGPS patient‐derived fibroblasts, and a mouse model of progeria to address those questions.

## 2 RESULTS

### 2.1 Inhibiting PLA2R1 expression overcomes progerin‐induced premature senescence

**Figure 1:**
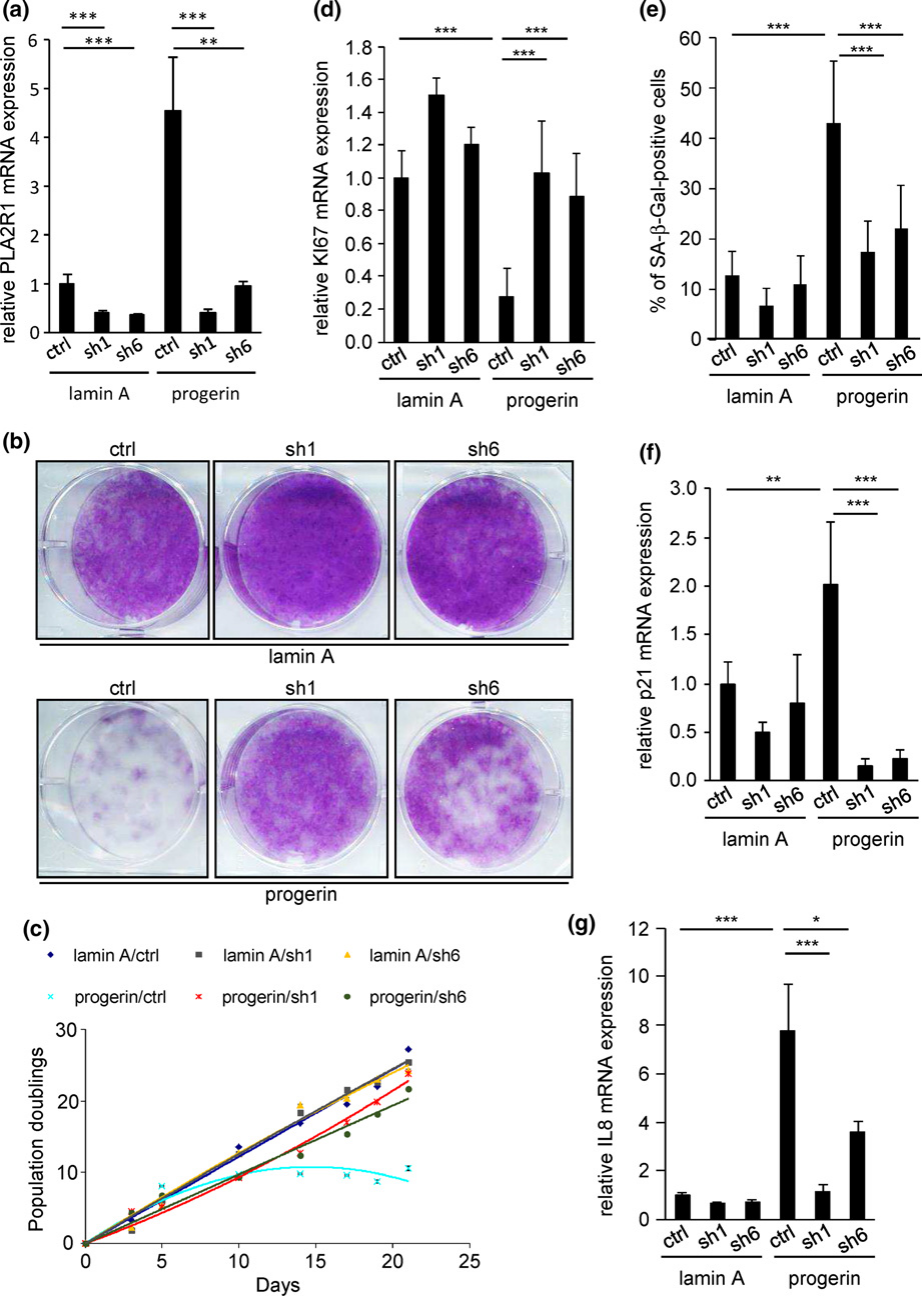
Phospholipase A2 receptor (PLA2R1) contributes to progerin‐induced senescence. MRC5 cells were infected with retroviral vectors encoding the indicated genes and shRNAs and selected. (a) Fifteen days after selection, RNA was isolated, reverse‐transcribed, and PLA2R1 transcripts were quantified by qPCR and normalized against ACTB levels. (b) Similar amounts of selected cells were seeded after the end of selection; cells were fixed 8 days later and stained with crystal violet. (c) At each passage, cells were counted and the number of population doublings was calculated, and the same number of cells was reseeded. (d) RNA was isolated 15 days after the end of the selection, reverse‐transcribed, and Ki67 transcript levels were assessed by quantitative PCR and normalized against ACTB levels. (e) Twelve days after the end of the selection, cells were stained for SA‐β‐Gal activity, and the percentage of SA‐β‐Gal‐positive cells was calculated. (f, g) RNA was prepared 15 days after the end of the selection, and RT–qPCR were performed against p21 (CDKN1A) or IL‐8. Results were normalized against ACTB. All results in this figure are representative of at least three independent experiments. Error bars indicate *SD*s of triplicate measurement. Statistical analysis was performed with Student's *t* test (*p < 0.05; **p < 0.01; ***p < 0.005).

Hutchinson–Gilford progeria syndrome is caused by the expression of progerin, a truncated form of lamin A (Goldman et al., 2004). To study the role of PLA2R1 in progerin‐induced senescence, we used normal human fibroblasts overexpressing progerin; control cells expressed lamin A. As expected, the proteins were localized in the nucleus and progerin was functionally active as it altered nuclear shape and we observed that PLA2R1 increased upon progerin expression (Supporting Information Figure S1a,b and Figure 1a). Next, we knocked down PLA2R1 expression using two different shRNA sequences (Figure 1a and Supporting Information Figure S1b). Constitutive expression of progerin resulted in proliferation arrest as judged by reduced number of cells observed using crystal violet staining (Figure 1b) and growth curves (Figure 1c) and reduced expression of the proliferation marker Ki67 (Figure 1d), and increased frequency of SA‐β‐Gal‐positive cells (Figure 1e and Supporting Information Figure S1c) and increased expression of p21 (CDKN1A) and the SASP component IL‐8 (Figure 1f,g). Knockdown of PLA2R1 with two independent shRNAs abolished all these hallmarks of cellular senescence (Figure 1b–g and Supporting Information Figure S1c). We conclude that PLA2R1 mediates premature senescence induced by the constitutive expression of progerin.

### 2.2 Loss of PLA2R1 extends replicative potential of HGPS patient‐derived cells

**Figure 2:**
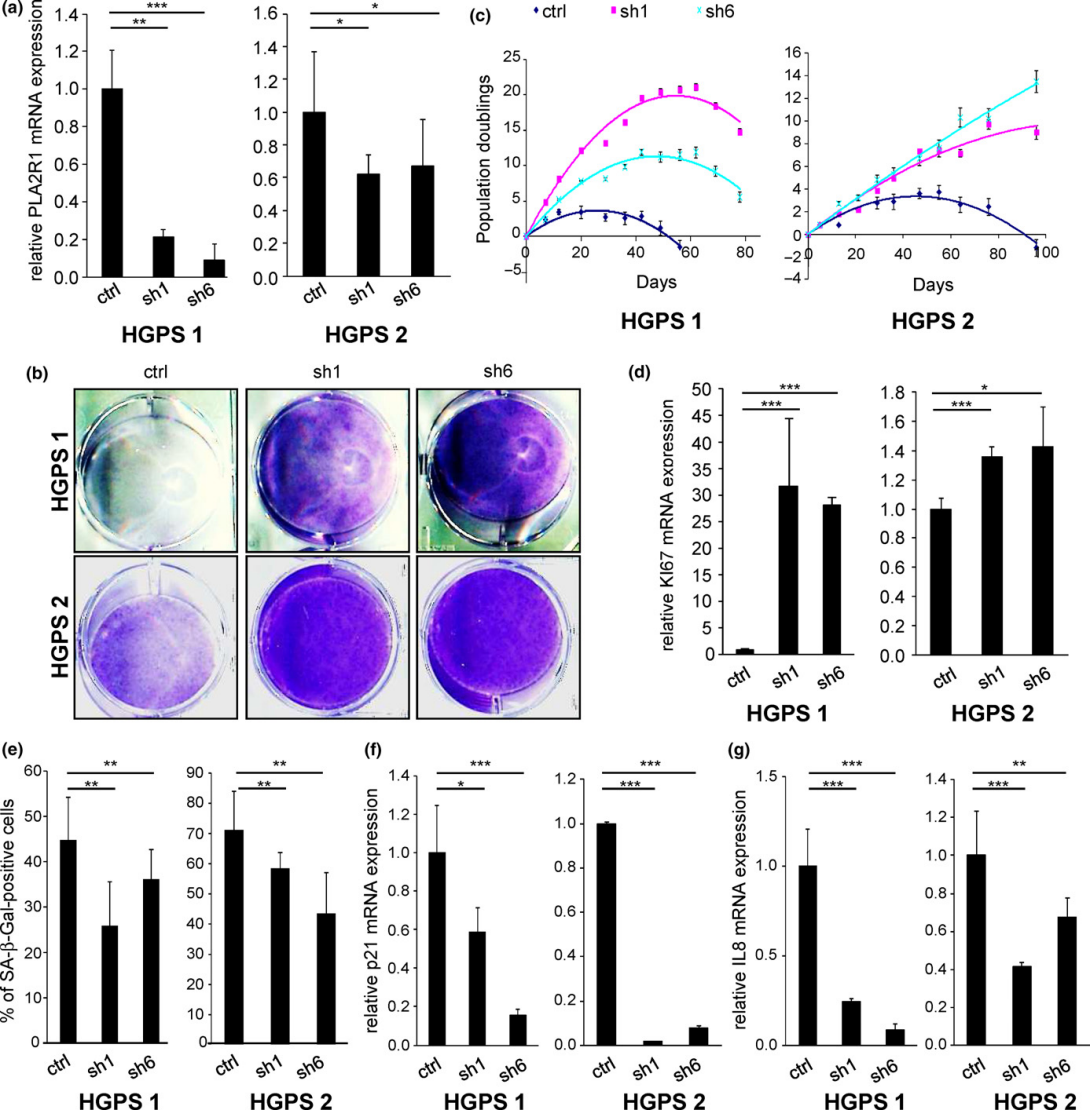
Loss of phospholipase A2 receptor (PLA2R1) extends the lifespan of Hutchinson–Gilford progeria syndrome (HGPS)‐derived cells. HGPS‐derived fibroblasts (HGPS 1 and 2) were infected with retroviral vectors encoding a scrambled or two different shRNAs directed against PLA2R1 and selected. (a) Twelve days after infection, RNAs were extracted reverse‐transcribed, and qPCR was performed against PLA2R1 and ACTB for normalization. (b) Five days after infection, the same amounts of cells were seeded. Twelve days later, cells were fixed and stained using crystal violet. (c) Population doubling was calculated at each passage, and the same number of cells was reseeded. (d) The levels of the proliferation marker Ki67 were quantified by qPCR and normalized against ACTB levels 12 days after infection. (e) Cells were stained 8 days after infection for SA‐β‐Gal activity, and the number of SA‐β‐Gal‐positive cells was counted for each condition. (f, g) Twelve days after infection, RNAs were prepared, and p21 or IL‐8 mRNA levels were measured by RT–qPCR and normalized against ACTB mRNA. All results in this figure are representative of at least two independent experiments. Error bars indicate *SD*s of triplicate measurement. Statistical analysis was performed with Student's *t* test (*p < 0.05; **p < 0.01; ***p < 0.005).

Primary dermal fibroblasts derived from patients with HGPS have a limited replicative potential compared to fibroblasts from healthy people (Eriksson et al., 2003). We introduced control or two different shRNA directed against PLA2R1 and verified PLA2R1 knockdown in two independent HGPS‐derived cells (Figure 2a). PLA2R1 knockdown extended the ability of these cells to proliferate, reduced the percentage of SA‐β‐Gal‐positive cells, and reduced p21 and IL‐8 expression (Figure 2b–g). In the same time frame of this experiment, we used control dermal fibroblasts without mutation in *LMNA* gene. These cells did not display premature senescence, and they were not impacted by the knockdown of PLA2R1 (Supporting Information Figure S2). Thus, PLA2R1 contributes to the reduced replicative potential of cells derived from patients with HGPS.

### 2.3 Loss of PLA2R1 reverts accumulation of misshapen nuclei

**Figure 3:**
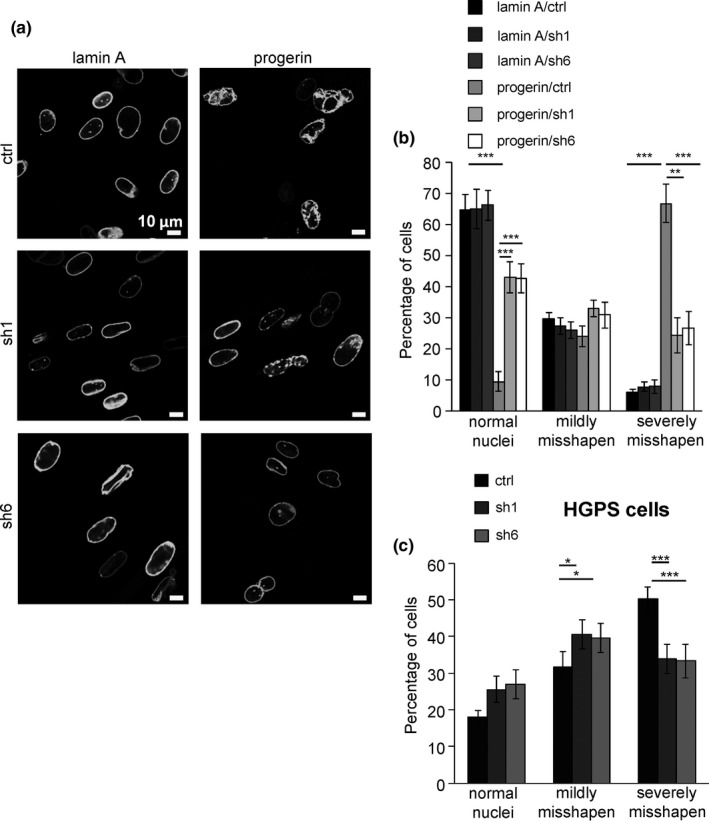
Phospholipase A2 receptor (PLA2R1) promotes progerin‐induced misshapen nuclei. (a, b) MRC5 cells were infected with retroviral vectors encoding lamin A‐GFP or progerin‐GFP together with control or PLA2R1 shRNA sequences. Ten days after selection, cells were analyzed by confocal microscope. Representative pictures are shown in panel a, and counting of normal, mildly misshapen, and severely misshapen nuclei was performed and is displayed in panel b. (c) Ten days after the end of the selection, immunofluorescence using a lamin A/C antibody was performed in HGPS‐derived fibroblasts encoding either control or shRNA directed against PLA2R1. The same classification as in b was used. Around 200 nuclei were counted for each condition (mean ± *SEM*s). The experiments shown are representative of at least three biological repeats. Statistical analysis was performed with Student's *t* test (*p < 0.05; **p < 0.01; ***p < 0.005).

Hutchinson–Gilford progeria syndrome patient‐derived cells exhibit a high frequency of misshapen nuclei which may contribute to disease pathogenesis (Goldman et al., 2004). Therefore, we next determined the impact of inhibiting PLA2R1 expression on nuclear shape in normal human fibroblasts constitutively expressing progerin and in HGPS cells stained with antibodies to lamin A/C. As expected, progerin expression led to an increase in the frequency of severely misshapen nuclei, and this increase was largely reverted upon PLA2R1 knockdown (Figure 3a,b). Similar results were obtained in HGPS cells (Figure 3c). These results demonstrate that the absence of PLA2R1 sustains normal nuclear shape in the presence of progerin.

### 2.4 PLA2R1 loss reduces defects in a mouse model of progeria

**Figure 4:**
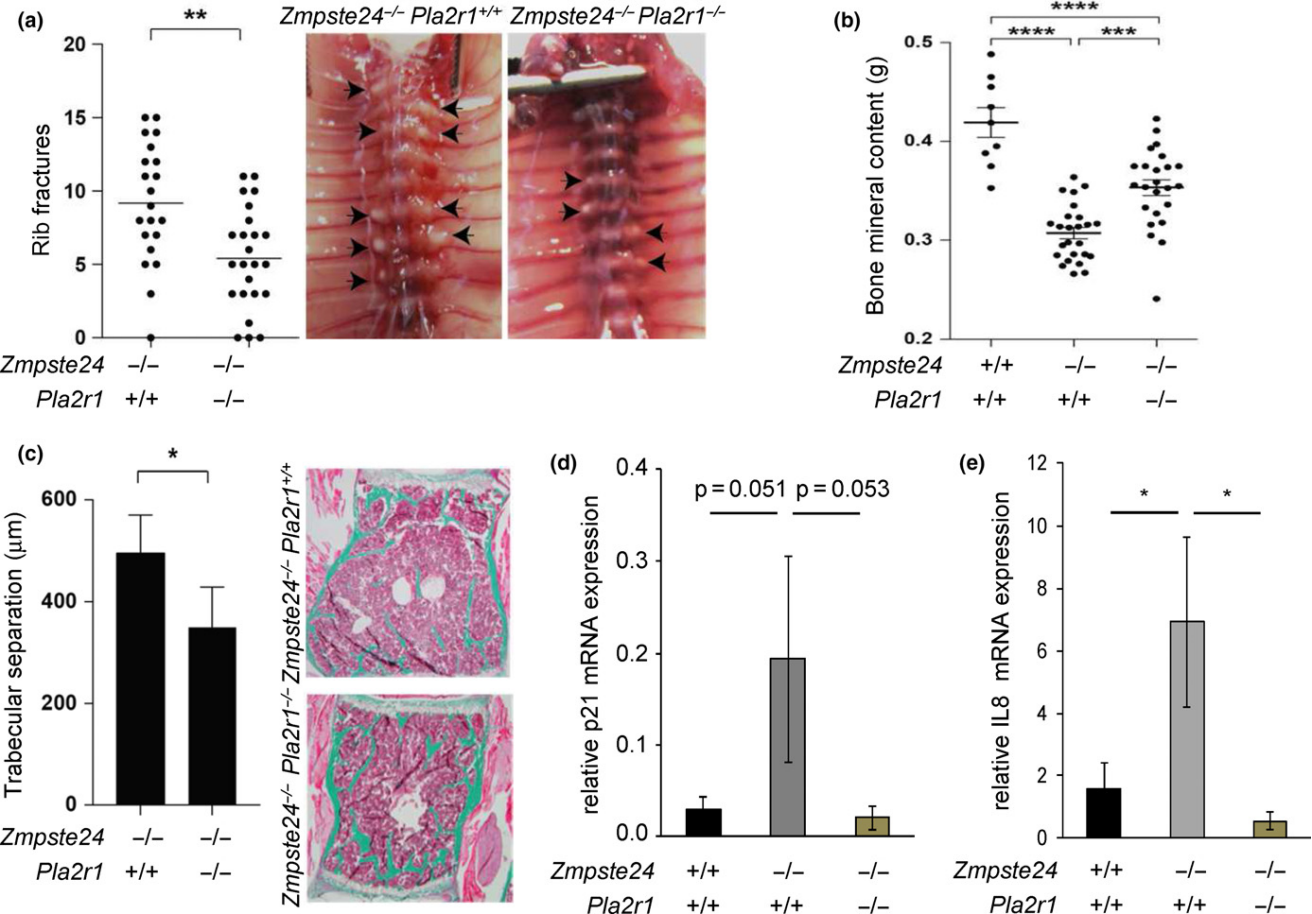
Loss of *Pla2r1* partially rescues defects in a murine HGPS model. (a) Number of rib fractures in *Zmpste24^−/−^Pla2r1^+/+^* (*n* = 21) and *Zmpste24^−/−^Pla2r1^−/−^* (*n* = 24) mice. Ventral view of spinal columns from *Zmpste24^−/−^Pla2r1^+/+^* and *Zmpste24^−/−^Pla2r1^−/−^*mice. Arrowheads indicate rib cage calluses, and signs of rib fractures at costovertebral junctions. (b) DEXA analyses of bone from 12‐ to 15‐week‐old mice. (c) Trabecular separation of L4 vertebrae (*n* = 5 per genotype, mean ± *SD*s) and representative Masson–Goldner trichrome‐stained sections of L4 vertebrae. (d, e) Bone mRNA levels of p21 and IL‐8 (*n* ≥ 3 per genotype) after normalization against ACTB levels (mean ± *SEM*s)

We next determined whether PLA2R1 contributes to progeria‐like phenotypes *in vivo*. For this, we used *Zmpste24*‐deficient mice which display accumulation of prelamin A, misshapen nuclei, and some hallmarks of progeria, including prominent bone alterations manifested as multiple rib fractures and reduced bone mineral content (Bergo et al., 2002; Figure 4a,b). Although knockout of *Pla2r1* did not change lifespan (Supporting Information Figure S3a), it reduced the number of rib fractures and increased bone mineral content (Figure 4a,b) and it improved grip strength (Supporting Information Figure S3b). Moreover, knockout of *Pla2r1* reduced trabecular separation in vertebral bone of *Zmpste24*‐deficient mice, as judged by histomorphometric analyses (Figure 4c). Interestingly, we found higher levels of the p53 target and senescence marker p21 as well as of IL‐8 senescence marker mRNA in bone of *Zmpste24*‐deficient mice than in wild‐type and that knockout of *Pla2r1* normalized those levels (Figure 4d,e). We conclude that loss of *Pla2r1* in a mouse model of progeria reduces bone alterations and signs of cellular senescence.

### 2.5 Loss of PLA2R1 reduces farnesyl diphosphate synthase expression and thereby improves nuclear shape abnormalities and promotes senescence escape

**Figure 5:**
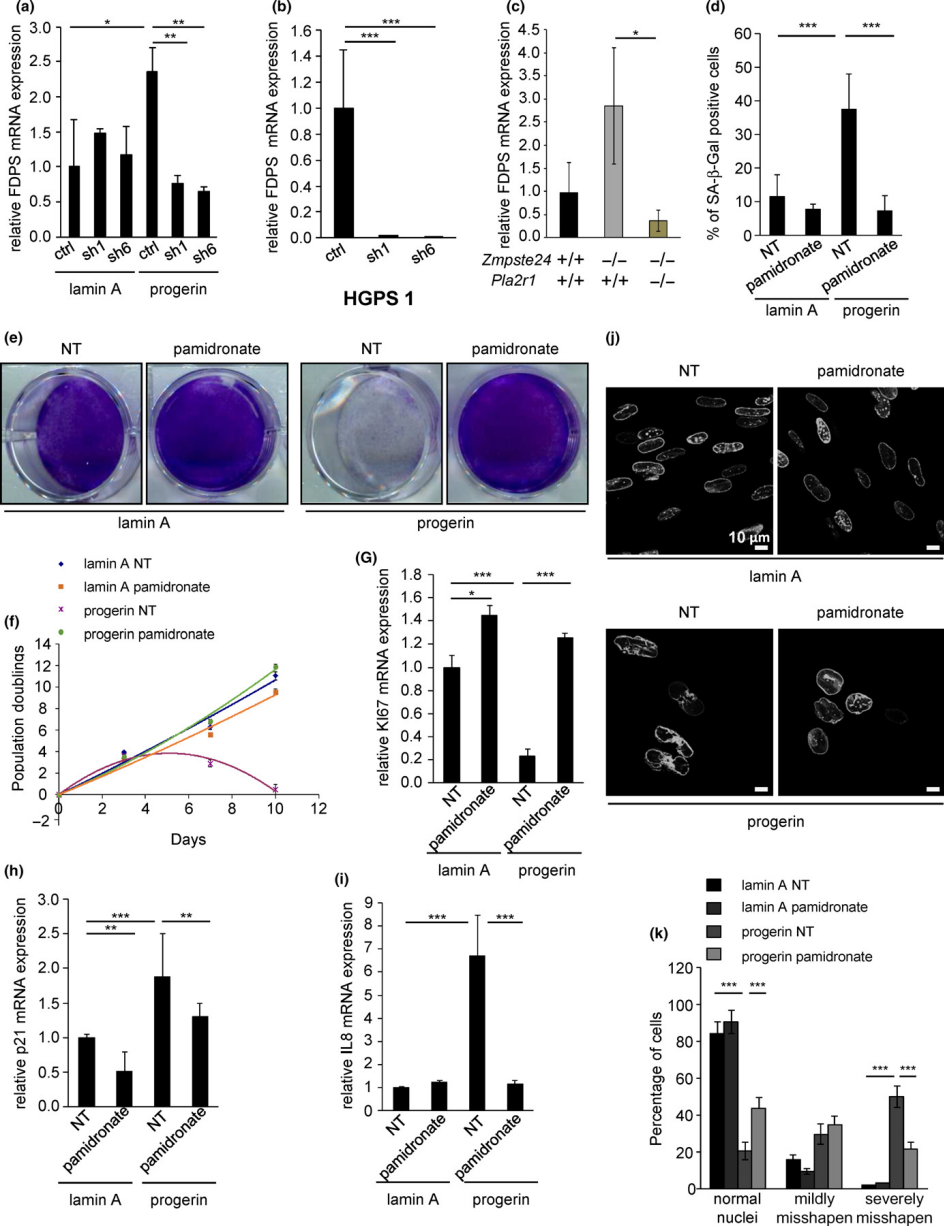
A progerin/PLA2R1 pathway increases FDPS expression and thereby induces senescence and misshapen nuclei. (a, b) Fifteen days after selection, RNAs were extracted from MRC5 or HGPS‐derived cells, and mRNA levels of FDPS were quantified by RT–qPCR for each indicated condition. Results were normalized against ACTB levels. (c) RNA was extracted from the bone of mice of the indicated genotypes before RT–qPCR experiments to measure FDPS levels. FDPS levels were normalized against ACTB levels. The number of mice per group ranged from 3 to 5. (d–k) MRC5 cells were infected with retroviral vectors encoding lamin A‐GFP or progerin‐GFP. After selection and seeding the same quantity of cells, cells were treated every 3 days with 5 µM pamidronate (a bisphosphonate FDPS inhibitor). (d) After 15 days of treatment, cells were stained for SA‐β‐Gal activity, and the percentage of SA‐β‐Gal‐positive cells was calculated. (e) Eight days after selection, the same amounts of cells were seeded. Ten days later, cells were fixed and stained with crystal violet. (f) At each passage, cells were counted and the number of population doublings was calculated, and the same number of cells was reseeded. (g–i) Fifteen days after selection, RNA was prepared, reverse‐transcribed, and indicated transcript levels were quantified by qPCR and normalized against ACTB levels. (j, k) Fourteen days after selection, cells were analyzed by confocal microscopy. Representative pictures are shown, and counting of normal, mildly misshapen, and severely misshapen nuclei was performed. The experiments shown are representative of at least three biological repeats. Statistical analysis was performed with Student's *t* test (*p < 0.05; **p < 0.01; ***p < 0.005).

Farnesyl diphosphate synthase (FDPS) may promote the formation of misshapen nuclei upon progerin expression, and it is also a target of bisphosphonate drugs which are used for clinical trials against HGPS (Gordon et al., 2016; Varela et al., 2008). We found increased FDPS expression in progerin‐expressing cells, in human HGPS cells, and in mouse model (Figure 5a–c). The loss of PLA2R1 normalized FDPS expression in all three conditions (Figure 5a–c). To determine whether the reduced FDPS expression contributed to senescence escape and improved nuclear shape in PLA2R1‐targeted cells, we used pamidronate, a bisphosphonate drug that inhibits FDPS activity. Indeed, incubating progerin‐expressing cells with pamidronate reduced senescence‐associated β‐galactosidase (SA‐β‐Gal) activity; increased cell proliferation and Ki67 expression; and reduced p21 and IL‐8 expression to levels observed in control cells (Figure 5d–i). Moreover, pamidronate reduced the frequency of misshapen nuclei (Figure 5j,k). These results suggest that reduced FDPS activity mediates the effects of PLA2R1 knockdown on progerin‐induced senescence and misshapen nuclei.

### 2.6 Dominant‐negative p53 reduces FDPS expression and improves nuclear shape

**Figure 6:**
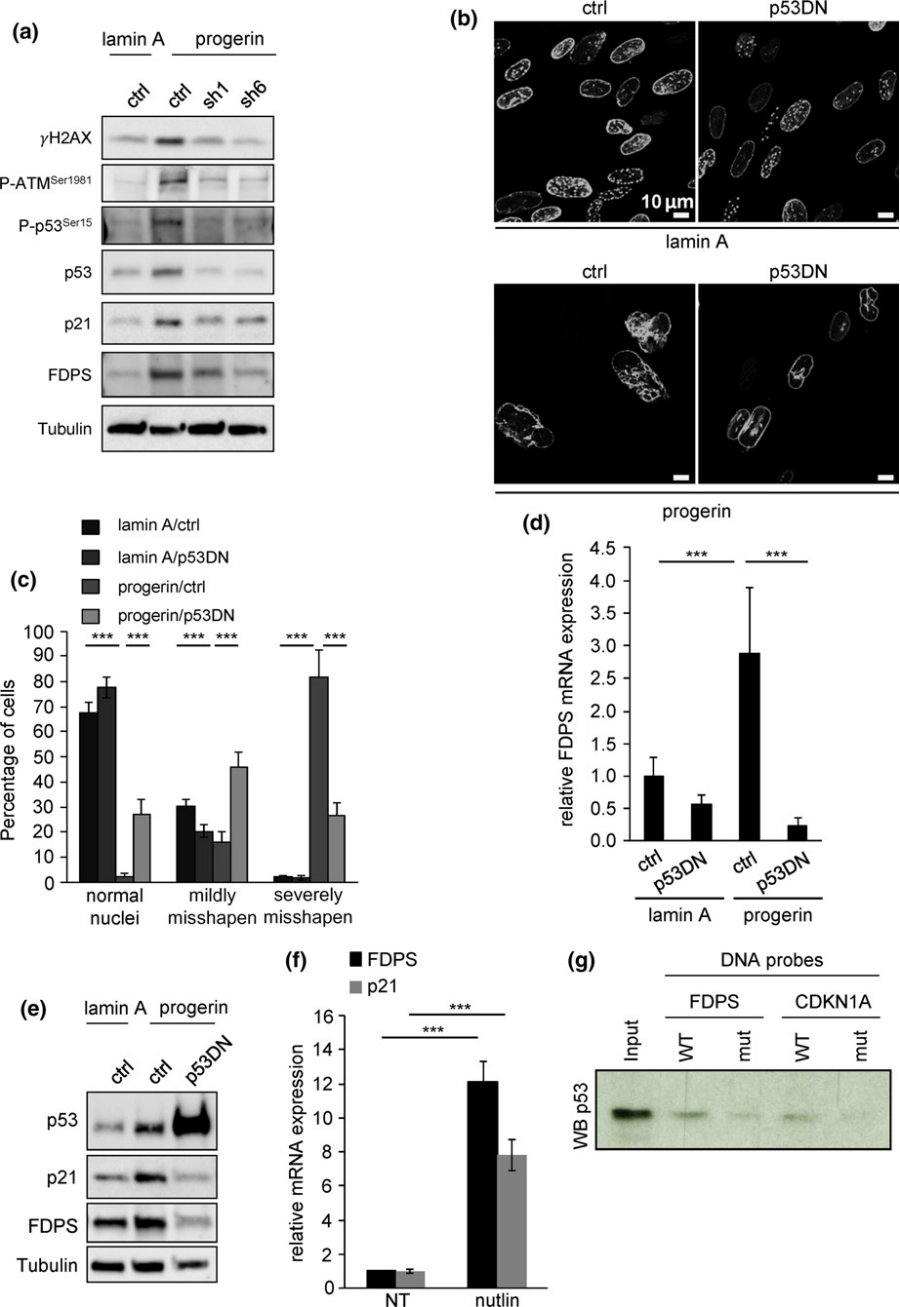
p53 promotes FDPS expression and misshapen nuclei in progerin‐expressing cells. (a) MRC5 cells infected with retroviral vectors encoding lamin A or progerin together with or without a retroviral vector encoding shRNA directed against PLA2R1. Fifteen days after selection, protein extracts were prepared and analyzed using antibodies targeting the indicated proteins. (b–e) MRC5 cells were infected with retroviral vectors encoding lamin A or progerin together with or without a retroviral vector encoding a dominant‐negative form of p53 (p53DN). (b) Twelve days after selection, photographs of nuclei and (c) quantification of normal, mildly misshapen, and severely misshapen nuclei were performed. (d) Ten days after infection and selection, RNAs were prepared, reverse‐transcribed, and FDPS transcripts were quantified by qPCR. Results were normalized against ACTB mRNA levels. (e) Twelve days after selection, protein samples were prepared. Samples were analyzed by immunoblot using antibodies targeting the indicated proteins. (f) MRC5 cells were treated with nutlin‐3, and 5 days later, RT–qPCR against FDPS and p21 was performed. Results were normalized against ACTB mRNA levels. (g) DNA pulldown assay showing p53 binding to a DNA motif in FDPS promoter. A lysate of MRC5 primary human fibroblasts treated with nutlin‐3 was incubated with FDPS probe or CDKN1A probe, harboring either a wild‐type (WT) p53‐binding motif or a mutant one with two base substitutions (mut). p53 binding was assessed by western blot. The experiments shown are representative of at least two biological repeats. Statistical analysis was performed with Student's *t* test (*p < 0.05; **p < 0.01; ***p < 0.005).

We showed earlier that PLA2R1 knockdown causes senescence escape by reducing DNA damage and p53 pathway activation (Augert et al., 2009). p53 also contributes to progerin‐induced senescence, and it may regulate FDPS expression in cancer cells (Kudlow, Stanfel, Burtner, Johnston, & Kennedy, 2008; Laezza et al.., 2015). However, whether p53 affects nuclear shape and whether it regulates FDPS expression in noncancer cells is not clear. As expected, progerin increased P‐ATM and γH2AX DNA damage marks (Figure 6a), p53 phosphorylation (Figure 6a), the p53 transcriptional target p21 (Figures 1f, 5h, and 6a), and FDPS (Figures 5a and 6a), and these inductions were abolished upon PLA2R1 knockdown (Figure 5a). Expressing a dominant‐negative p53 (p53DN) overcame progerin‐induced senescence: p53DN increased proliferation of progerin‐expressing cells and reduced p21 and IL‐8 expression (Supporting Information Figure S4). Similar to PLA2R1 inactivation, p53DN also reduced the frequency of misshapen nuclei and FDPS expression in progerin‐expressing cells (Figure 6b–e). Furthermore, p53 activation by nutlin‐3, a known activator of p53 (Vassilev et al., 2004), led to increased FDPS mRNA similar to the observed p21 increase and led to binding of p53 on the p53 binding site previously described on FDPS promoter in cancer cells (Laezza et al., 2015; Figure 6f–g). The results suggest that p53 contributes to progerin/PLA2R1‐induced misshapen nuclei and cellular senescence by upregulating FDPS expression.

## 3 DISCUSSION

In this study, we found that PLA2R1 mediates effects of progerin expression on cellular senescence and some premature aging hallmarks. The knockdown of PLA2R1 overcame the proliferation arrest and other characteristics of senescent cells (SA‐β‐Gal activity and increased IL‐8) induced by progerin, either ectopically or endogenously expressed in mutated cells. Because PLA2R1 participates in senescence induced by short telomeres, oncogene, and oxidative stress, the current results reinforce the concept that PLA2R1 is a master regulator of cellular senescence (Augert et al., 2009; Bernard, & Vindrieux, 2014; Vindrieux et al., 2013).

It has previously been suggested that FDPS could promote the formation of misshapen nuclei upon progerin expression, and it is also a target of bisphosphonate drugs which are used for clinical trials against HGPS (Gordon et al., 2016; Varela et al., 2008). Bisphosphonates target FDPS, and according to our results, FDPS levels increased upon progerin and prelamin A expression (i.e., in HGPS‐derived cells and *Zmpste24*‐deficient bone, respectively) and targeting FDPS with pamidronate overcame progerin‐induced senescence and misshapen nuclei. These results suggest that under normal conditions PLA2R1 contributes to progerin‐induced phenotypes by stimulating FDPS expression. Progerin was found to increase PLA2R1 levels. Even if we do not know how, induction of PLA2R1 in the context of replicative senescence has already been described (Augert et al., 2009), suggesting that PLA2R1 is induced and it participates to different types of senescence. PLA2R1 regulates senescence through induction of a mitochondrial program leading to ROS production, DNA damage, and the p53 pathway (Augert et al., 2009; Griveau et al., 2016). Progerin is able to induce DNA damage accumulation, eventually through ROS generation (Musich, & Zou, 2011; Richards, Muter, Ritchie, Lattanzi, & Hutchison, 2011; Wheaton et al., 2017). Our data support a role of PLA2R1 in progerin‐induced DNA damage as its knockdown reverts accumulation of DNA damage marks such as γH2AX and P‐ATM. DNA damage signaling leads to phosphorylation and activation of p53 transcriptional activity, both being observed in progerin‐expressing cells and reversed in PLA2R1 knockdown cells. p53 has been reported to impact progerin‐induced senescence (Kudlow et al., 2008) and once to bind to the FDPS promoter and to regulate FDPS expression in cancer cells (Laezza et al., 2015). Thus, it is conceivable that p53 downstream of progerin and PLA2R1 could mediate the increased FDPS expression. Accordingly, we found that p53 loss of function abolishes progerin‐induced senescence, misshapen nuclei, and FDPS increase, whereas its activation by nutlin‐3 induces FDPS expression suggesting the presence of a progerin/PLA2R1/p53/FDPS prosenescent axis in HGPS cells.

Beyond *in vitro* results, little was known on the potential role of PLA2R1 in regulating age‐related diseases, aging, or premature aging *in vivo*. Some HGPS and aging hallmarks are replicated in *Zmpste24*‐deficient mice due to loss of lamin A maturation (Bergo et al., 2002; Burtner, & Kennedy, 2010; Gordon, Rothman, Lopez‐Otin, & Misteli, 2014; Ibrahim et al., 2013). Our results using *Pla2r1* and *Zmpste24* double‐knockout mice support a functional role for PLA2R1 in controlling cellular senescence and some aging hallmarks. Indeed, the loss of *Pla2r1* partially rescues the decreased bone content, the increased rib fracture, and the increased senescence markers observed in the bone of the *Zmpste24* knockout mice. Our results thus reveal an inverse correlation between senescence markers and bone content and fragility, suggesting that the correlation might be functionally linked. We can thus speculate that loss of PLA2R1, by reducing senescence, could improve this mark of aging. Supporting this hypothesis, a causal role for senescent cells has recently been described in bone loss during aging as eliminating senescent cells or inhibiting JAK kinases, in old mice, delayed bone loss (Farr et al., 2017). We have previously reported that PLA2R1 exerts its prosenescent effects through JAK kinases reinforcing a potential link between PLA2R1, the mechanisms it controlled, and bone defects (Bernard & Vindrieux, 2014; Vindrieux et al., 2013). We can speculate that JAK kinase inhibitors could also be candidate drugs to fight premature aging, at least for the bone defects part.

The bisphosphonate family of drugs is used to fight bone loss during aging (Russell, Watts, Ebetino, & Rogers, 2008). Independently of this known effect, bisphosphonates also decrease misshapen nuclei in HGPS cells, which are proposed to participate in the disease, and trials using this drug have been set up for this reason (Gordon et al., 2016; Varela et al., 2008). Our results point out that bisphosphonate strongly impacts senescent phenotype reinforcing a potential functional link between bone fragility during aging and increase cellular senescence. It has also been reported that bisphosphonates can impact bone‐independent marks of aging like emphysema (Ueno et al., 2015), emphysema being thought to be regulated by cellular senescence (Taraseviciene‐Stewart, & Voelkel, 2008), further suggesting that FDPS/bisphosphonates can regulate cellular senescence and aging beyond their well‐known bone effects.

In conclusion, our results demonstrate that PLA2R1 could be a new factor that contributes to hallmark phenotypes of premature aging and suggest that it could constitute a novel target to impact some aging parameters.

## 4 MATERIALS AND METHODS

### 4.1 Cell culture and reagents

MRC5 normal human fibroblasts (ATCC, Manassas, VA, USA) and virus‐producing GP293 cells (Clontech, Mountain View, CA, USA) were cultured in Dulbecco′s modified Eagle′s medium (DMEM, Life Technologies, Carlsbad, USA) containing GlutaMAX and supplemented with 10% FBS (Sigma‐Aldrich, Saint Louis, USA) and 1% penicillin/streptomycin (Life Technologies). Dermal fibroblasts from patients with HGPS carrying the 1824 C > T mutation were obtained from the Progeria Research Foundation (HGADFN003; Peabody, MA) and the NIA Aging Cell Culture Repository (AG03199; New Jersey, USA); dermal fibroblasts from control patient without *LMNA* mutation were obtained from the NIA Aging Cell Culture Repository (AG03258; New Jersey, USA) and were cultured in DMEM supplemented with 20% FBS and 1% penicillin/streptomycin. Information of primary fibroblasts and their usage in the figures are displayed in Supporting Information Table S1. Cells were maintained at 37°C under a 5% CO_2_ atmosphere. Pamidronate (506600, Merck Millipore, Billerica, USA), a FDPS inhibitor, was used at 5 µM. Nutlin‐3 (Sigma‐Aldrich), a p53 inducer, was used at 5 µM.

### 4.2 Vectors, transfection, and infection

The following vectors were supplied by Addgene: pBABE‐puro‐GFP‐wt‐lamin A (#17662) and pBABE‐puro‐GFP‐progerin (#17663). PLA2R1‐shRNA‐encoding retroviral vectors have been previously described (Augert et al., 2009).

Virus‐producing GP293 cells were transfected with vectors using the GeneJuice reagent according to the manufacturer's recommendations (Merck Millipore). Cells were transfected with the VSVg (1 µg) and the retroviral vector of interest (5 µg). Two days after transfection, the viral supernatant was mixed with fresh medium (1/2) and hexadimethrine bromide (8 μg/ml; Sigma‐Aldrich), and was then used to infect target cells for 6 hr. One day postinfection, selection was started with neomycin (100 mg/ml), puromycin (500 ng/ml), or both.

### 4.3 RNA extraction, reverse transcription, and real‐time quantitative PCR

RNA was extracted with phenol–chloroform using Upzol (Dutscher, Brumath, France). The Maxima First cDNA Synthesis Kit (Life Technologies) was used to synthesize cDNA from 1 μg of total RNA. The reverse transcription (RT) reaction mixture was diluted 1/20 and used as cDNA template for quantitative PCR (qPCR) analysis. TaqMan qPCR analyses were carried out on a FX96 Thermocycler (Bio‐Rad, Hercules, USA). The PCR mixture contained TaqMan mix (Roche, Boulogne‐Billancourt, France), 200 nM of primers, the Universal Probe Library probe (100 µM) for the gene of interest (TaqMan Gene Expression Assays [Primers/probe]; Life technologies), and 1.67 μl cDNA template. Reactions were performed in triplicate. The relative amount of mRNA was calculated using the comparative Ct (ΔΔCT) method, following data normalization against ACTB for housekeeping genes. The PCR primers used for the qPCR are listed in Supporting Information Table S2.

### 4.4 Senescence‐associated β‐galactosidase analysis, crystal violet staining, and growth curves

To perform SA‐β‐Gal assays, cells were washed twice with PBS, fixed for 5 min in 2% formaldehyde/0.2% glutaraldehyde, rinsed twice in PBS, and incubated at 37°C overnight in SA‐β‐Gal solutions as described (Augert et al., 2009). For crystal violet staining, cells were seeded on 6‐ or 12‐well plates; staining was performed 8–10 days later as described (Augert et al., 2009). For growth curves, selected cells were seeded at the same density, split twice a week and counted; the population doubling was calculated at each passage.

### 4.5 Quantification of misshapen nuclei

Cells expressing lamin A‐GFP or progerin‐GFP vectors and HGPS patient‐derived and control cells were seeded in 8‐well chamber‐slide plates (Dutscher); 2 days later, immunofluorescence was performed using a lamin A/C antibody (sc‐6215; Santa Cruz Biotechnology). The cells were analyzed with a Zeiss LSM 780 NLO confocal microscope using a 63× oil‐immersion objective. Images were captured and processed using the Zen software (cropping, addition of scale bars). Nuclei were classified into three categories according to their shape: normal, mildly misshapen, or severely misshapen. Around 200 nuclei were counted for each condition.

### 4.6 Immunoblot

Twelve to fifteen days after retroviral infection with the indicated vectors, cells were directly lysed into Laemmli buffer supplemented with 10% β‐mercapto‐ethanol. The lysates were resolved on 12% or 4%–20% Mini‐PROTEAN gels (Bio‐Rad) and transferred to nitrocellulose membranes (0.22 µm; Bio‐Rad). The membranes were blocked with TBST–milk 5% for 1 hr and incubated with primary antibodies overnight at 4°C. Secondary antibodies were incubated for 1 hr at room temperature. Antibodies and dilutions used were as follows: p21 (C‐19, sc‐397, Santa Cruz Biotechnology, 1:500), p53 (DO‐1, sc‐126, Santa Cruz Biotechnology, 1:1,000), phospho‐p53Ser15 (9284, Cell Signaling, 1:500), α‐tubulin (T6199, Sigma‐Aldrich, 1:5,000), FDPS (HPA028200, Atlas Antibodies, 1:200), phospho‐ATM S1981 (ab81292, Abcam, 1:1,000), PLA2R1 (AMAB90772, Atlas Antibodies, 1:500), and phospho‐histone H2AX (2577S, Cell Signaling, 1/1,000). HRP‐conjugated secondary antibodies were anti‐mouse (115‐035‐003) and anti‐rabbit (111‐035‐003) from Jackson Laboratories.

### 4.7 Mice

Mice were maintained on a mixed background (C57BL/6 and SV129*). Pla2r1^−/−^*mice (Hanasaki, Yokota, Ishizaki, Itoh, & Arita, 1997) were bred with *Zmpste24^−/−^*mice (Bergo et al., 2002) to produce *Zmpste24^−/−^Pla2r1^−/−^*mice; littermate *Zmpste24^−/−^Pla2r1^+/+^* mice were used as controls. The ability of mice to hang for more than 30 s on to an upside‐down grid was assessed every week. Mice were considered having an impaired grip when they were unable to hang 2 weeks in a row. Mice were monitored daily. Mouse experiments were approved by the Research Animal Ethics Committee in Gothenburg.

### 4.8 Bone analyses

Whole‐body bone mineral content was determined by dual‐energy X‐ray absorptiometry (DXA) using a Lunar PIXImus densitometer (Wipro, GE Healthcare). Bone histomorphometry was used to analyze trabecular bone in an undecalcified lumbar vertebra (L4) stored in 70% EtOH (Erben, & Glosmann, 2012). Each sample was dehydrated in increasing concentrations of EtOH, defatted in xylene, and embedded in methyl methacrylate. The embedded sample was sectioned longitudinally in a coronal plane using an automated rotary microtome (Leica RM2265; Leica Microsystems, Wetzlar, Germany) and a tungsten‐carbide knife. Plastic 4‐μm sections were obtained from a standardized site of the vertebral body cavity. The sections were stained with Masson–Goldner trichrome, and trabecular separation was determined. In each section, the analysis of trabecular bone using OsteoMeasure 7 histomorphometry system (OsteoMetrics, Atlanta, GA, USA) was performed using a region of interest with the total area of 1.5 mm^2^.

### 4.9 DNA pulldown assay

MRC5 primary human fibroblasts were treated with 5 µM nutlin‐3. Five days later, cell lysate was prepared in HKMG buffer containing 10 mM Hepes pH 7.9, 100 mM KCl, 5 mM MgCl2, 10% glycerol, 0.5% NP‐40, 1 mM DTT, and protease inhibitors (Complete EDTA‐free, Roche) and was precleared with preequilibrated streptavidin‐coupled Dynabeads (Invitrogen). Annealed pairs of complementary oligonucleotides with the sense oligonucleotide biotinylated at the 5′ end (Sigma) were used for DNA pulldown. Oligonucleotide sequences are listed in Supporting Information Table S3. These DNA probes were incubated overnight at 4°C with the cell lysate. DNA‐bound proteins were then collected by incubation for 1 hr at 4°C with streptavidin‐coupled Dynabeads (Invitrogen), washed four times in HKMG buffer, and separated by SDS‐PAGE. p53 was detected by western blot (p53 antibody, DO‐1, sc‐126; Santa Cruz).

### 4.10 Statistical analysis

Values are mean ± *SD* or *SEM* as indicated in the figure legend. Statistical analyses were performed using Student's *t* test or one‐way ANOVA with Bonferroni's post hoc test when comparing three or more groups (*p < 0.05; **p < 0.01; ***p < 0.005).

## CONFLICT OF INTEREST

None declared.

## AUTHOR CONTRIBUTIONS

A.G. and C.W. designed and performed the experiments, analyzed the results, and wrote the manuscript. D.B. and M.O.B. supervised, conceived, and designed the experiments; analyzed the results; and wrote the manuscript. B.L.C., D.V.Z., S.D., M.W., N.M., J.M., and D.V. performed the experiments and/or analyzed the results. All authors reviewed or edited the manuscript and approved its final version.
