# Supplementary material for: Targeting the phospholipase A2 receptor ameliorates premature aging phenotypes
Source: Aging Cell. 2018 Sep 14;17(6):e12835. doi: 10.1111/acel.12835 (PMC6260922; doi:10.1111/acel.12835)
Supplement: Supplementary file 1 [file ACEL-17-e12835-s001.pdf]

Sup Figure 1

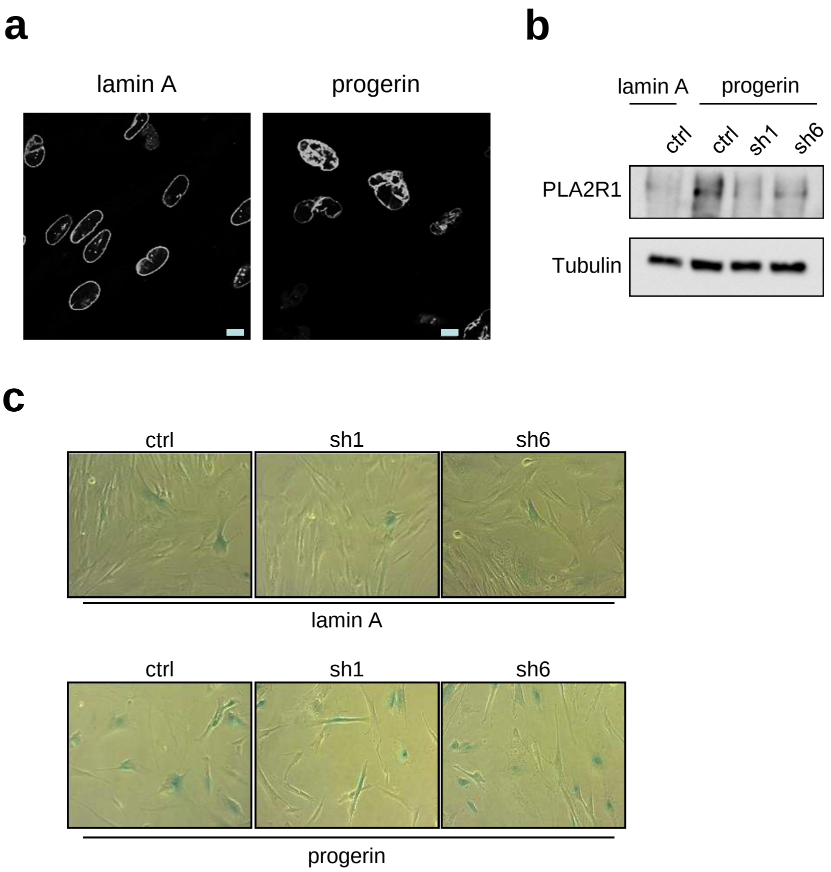

Supplementary Figure 1 (a) Progerin-induced misshapen nuclei in normal human MRC5 fibroblasts. MRC5 cells were infected with lamin A-GFP or progerin-GFP encoding retroviral vectors. Confocal experiment was performed ten days after selection and representative images are shown. (b) MRC5 cells infected with retroviral vectors encoding lamin A or progerin together with or without a retroviral vector encoding shRNA directed against PLA2R1. Fifteen days after selection, protein extracts were prepared and analyzed using PLA2R1 and tubulin antibodies. (c) Representative photos of the SA-b-Gal staining used for the quantification displayed Figure 1e.

Sup Figure 2

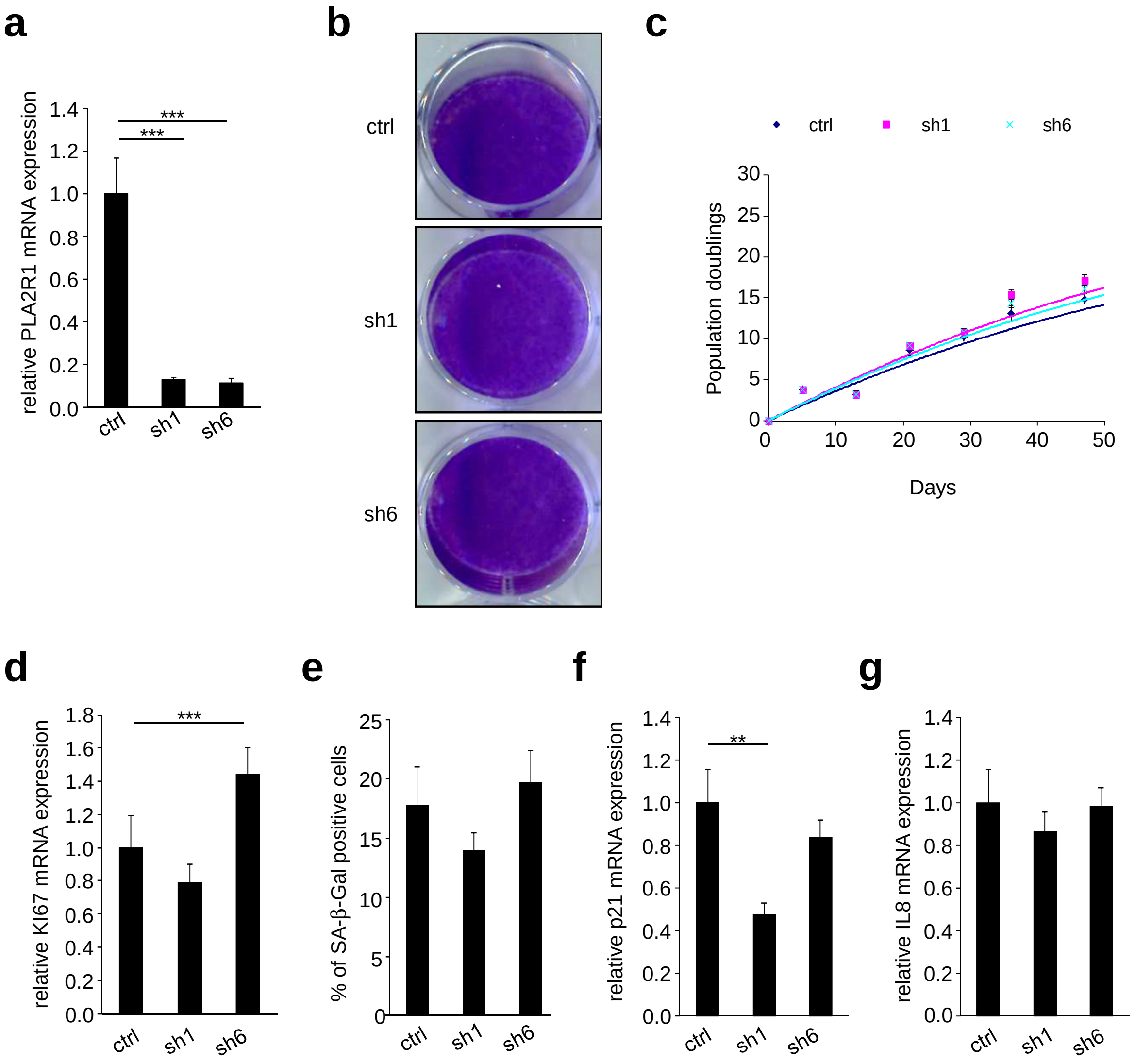

Supplementary Figure 2 Behavior of control dermal fibroblasts without LMNA gene mutation. Cells were infected with retroviral vectors encoding control or two different shRNAs directed against PLA2R1. (a) Twelve days after infection, RNA was prepared. PLA2R1 knockdown was verified by RTqPCR. PLA2R1 transcript levels were normalized against ACTB levels. (b) Twelve days after seeding the same amount of cells, cells were fixed and stained using crystal violet. (c) At each passage, cells were counted and the number of population doublings was calculated at each passage and the same number of cells was re-seeded. (d) RNA was prepared 12 days after infection. KI67 proliferation marker levels were quantified using RTqPCR after normalization to ACTB levels. (e) SA-β-Gal assays were performed 8 days after infection and the number of SA-β-Gal positive cells counted. (f-g) RNA was prepared 12 days after infection. p21 (CDKN1A) or IL8 mRNA levels were measured by RTqPCR and theirs levels normalized to the level of ACTB mRNA. Error bars indicate SDs of triplicate measurement. Statistical analysis was performed with the Student's t-test (* $p < 0.05$; ** $p < 0.01$; *** $p < 0.005$).

Sup Figure 3

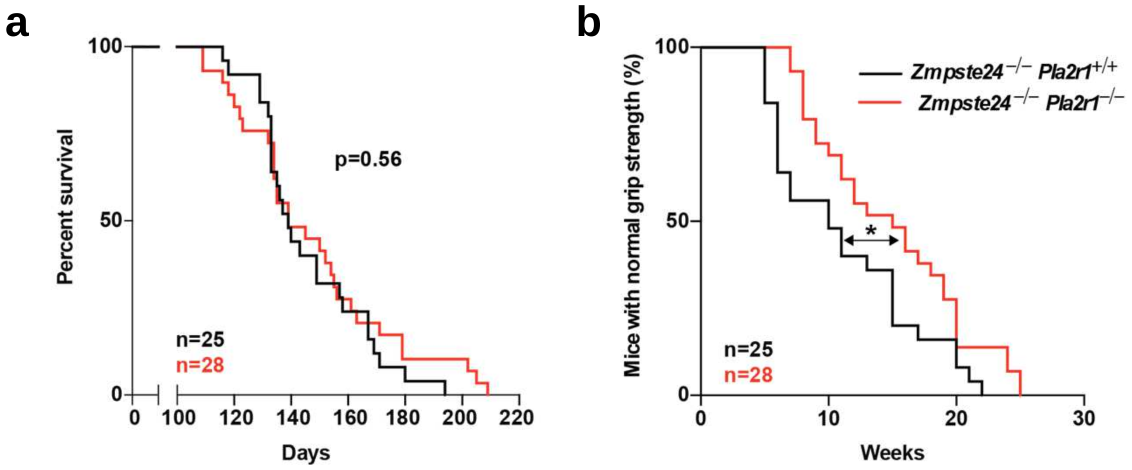

Supplementary Figure 3 Loss of PLA2R1 improves grip strength but not survival in Zmpste24-/- mice. (a) Kaplan-Meier plot showing survival of Zmpste24-/- Pla2r1 +/+ (n=25) and Zmpse24-/-Pla2r1 -/- (n=28) mice. (b) Kaplan-Meier plot showing the percentage of Zmpste24-/- Pla2r1 +/+ (n=25) and Zmpse24-/- Pla2r1 -/- (n=28) mice with normal grip strength. (*P <0.05).

Sup Figure 4

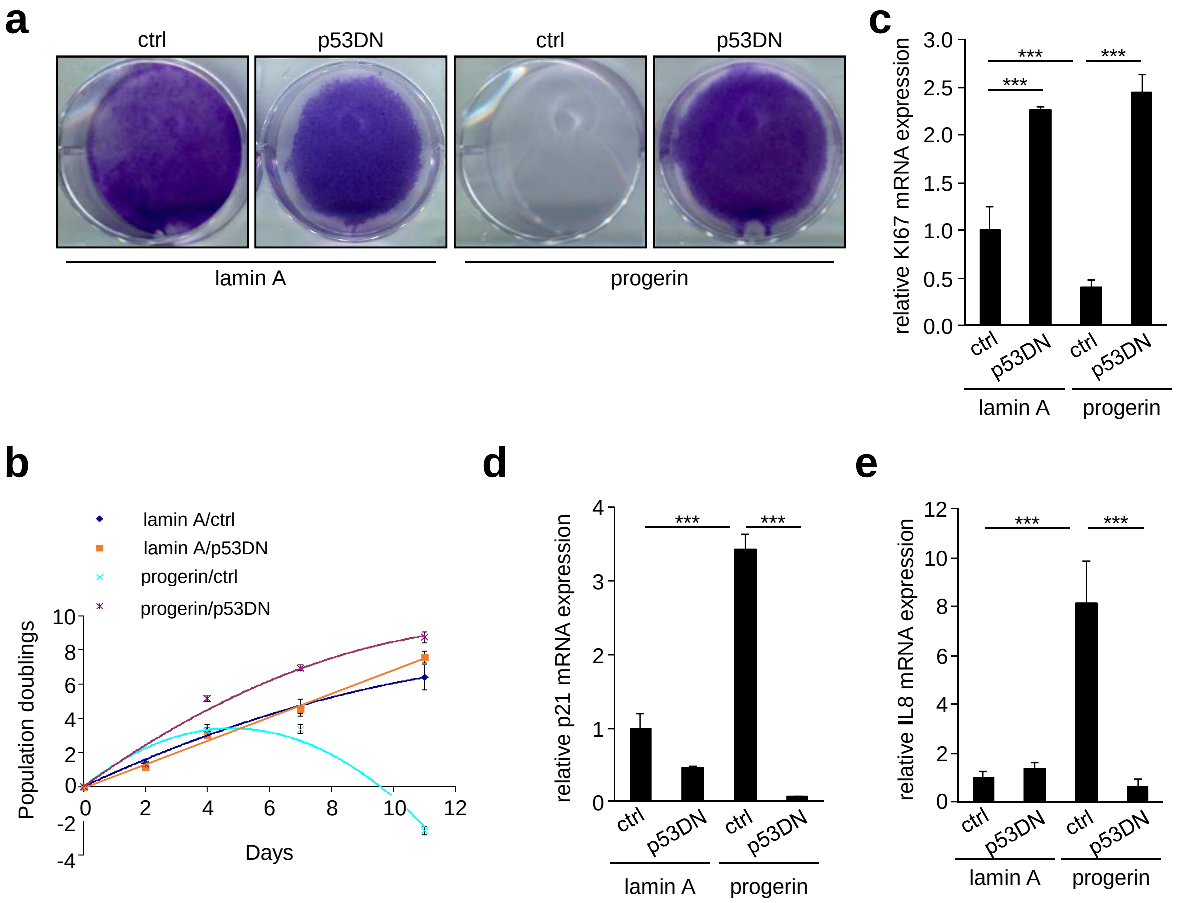

Supplementary Figure 4 Loss of p53 function bypasses progerin-induced senescence. MRC5 cells were infected with retroviral vectors encoding the indicated products and selected. (a) Similar amount of cells were seeded. Eight days later, cells were fixed and stained using crystal violet. (b) At each passage, cells were counted and the number of population doublings calculated and the same number of cells was re-seeded. (c-e) RNAs were prepared, reverse transcribed, and indicated transcript levels were quantified by qPCR and normalized against ACTB levels. The experiments shown are representative of at least 3 biological repeats. Error bars indicate SDs of triplicate measurement. Statistical analysis was performed with the Student's t-test (*** $p < 0.005$).

Sup Table 1

Name	Cell type (origin)	Source	LMNA mutation	Name in the Figures	Figure using these cells
MRC5	Human fibroblasts (lung)	ATCC	WT	MRC5	Figure 1a-g; Figure 3a-b; Figure 5a, 5d-k; Figure 6a-h
HGADFN003	Human fibroblasts from HGPS patient (skin)	Progeria Research Foundation	1824 C > T	HGPS 1	Figure 2a-g; Figure 3c; Figure 4b
AG03199	Human fibroblasts from HGPS patient (skin)	NIA Aging Cell Culture Repository	1824 C > T	HGPS 2	Figure 2a-g
AG03258	Human fibroblasts from control patient (skin)	NIA Aging Cell Culture Repository	WT	control	Supplementary Figure 2

Table 1. Information of primary fibroblasts used and their usage.

Sup Table 2

	Name	Primer L sequence	Primer R sequence	UPL
human primers	**Actin**	ATTGGCAATGAGCGGTTC	GGATGCCACAGGACTCCAT	11
	**PLA2R1**	CATAAGTGGATTTCTTATGGGTCA	CCCTTTGATTGTATGCAAATCTT	22
	**KI67**	TCAAGGAACTGATTCAGGAGAAG	GTGCACTGAAGAACACATTTCC	32
	**IL8**	AGACAGCAGAGCACACAAGC	ATGGTTCCTTCCGGTGGT	72
	**p21**	TCACTGTCTTGTACCCTTGTGC	GGCGTTTGGAGTGGTAGAAAT	32
	**FDPS**	GAGTACCCGCCAACAAGC	ATCTCAACCAGCGGGACA	15
murine primers	**Actin**	CTAAGGCCAACCGTGAAAAG	ACCAGAGGCATACAGGGACA	64
	**IL8**	TTCTGAGCTTGCTGGGAAAC	GGGTCCTTCGCCTGTATAAGA	9
	**p21**	TGCGCTTGGAGTGATAGAAA	AACATCTCAGGGCCGAAA	16
	**FDPS**	TCCTTCTGCCCATAATTCTCC	GGTGGTTCAGTGTCTGCTACG	91

Table 2. Human and murine primers and UPL probes used for quantitative PCR experiments.

Sup Table 3

Oligonucleotide	Oligonucleotide
FDPS_WT_F	AACCTACCAAAAC**CAAG**ATGGCAA**CAAG**AGTGACCTCTGG
FDPS_WT_R	CCAGAGGTCACTCTTGTTGCCATCTTGGTTTTGGTAGGTT
FDPS_mut_F	AACCTACCAAAACTAACATGGCAATAACAGTGACCTCTGG
FDPS_mut_R	CCAGAGGTCACTGTTATTGCCATGTTAGTTTTGGTAGGTT
CDKN1A_WT_F	TGGCCATCAGGAA**CATG**TCCCAA**CATG**TTGAGCTCTGGCA
CDKN1A_WT_R	TGCCAGAGCTCAACATGTTGGGACATGTTCCTGATGGCCA
CDKN1A_mut_F	TGGCCATCAGGAATATCTCCCAATATCTTGAGCTCTGGCA
CDKN1A_mut_R	TGCCAGAGCTCAAGATATTGGGAGATATTCCTGATGGCCA

Bold: core C(A/T)(T/A)G p53-binding motif on the forward (F) oligonucleotide.
Underlined: base substitutions in the core p53-binding motif (CXXG to TXXC) on the forward (F) oligonucleotide.

Table 3. Oligonucleotide sequences used by DNA pull down.
